# Westerlund and Narayan predictability test: Step-by-step approach using COVID-19 and oil price data

**DOI:** 10.1016/j.mex.2020.101201

**Published:** 2020-12-25

**Authors:** Susan Sunila Sharma

**Affiliations:** Centre for Financial Econometrics & Department of Finance, Faculty of Business and Law, Deakin University, 221 Burwood Highway, Burwood, Victoria 3125, Australia

**Keywords:** Predictability, COVID-19, Oil price

## Abstract

In this note, we provide a step-by-step approach of Westerlund and Narayan (WN, 2012, 2015) predictability test using COVID-19 and oil price data. This is an important exercise because the WN model addresses three salient features of time series data, namely persistency, endogeneity and heteroskedasticity. We consider COVID-19 and oil price data as predictors of stock market returns for four Asian countries to demonstrate the applicability of the WN (2012, 2015) predictability approach.

• This note demonstrates a step-by-step approach of the WN (2012, 2015) predictability test.
• WN model accommodates three salient features of time-series data, namely persistency, endogeneity, and heteroskedasticity.
• COVID-19 and oil price does not significantly predict stock returns of Japan, Russia, and Singapore (except in the case of South Korea).

## Graphical abstract

**Figure:**
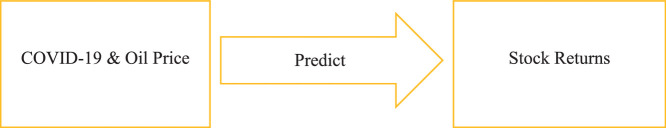

**Specifications Table**

**Table:** 

Subject Area:	Economics and Finance
More specific subject area:	*Financial stock market and forecasting*
Method name:	Westerlund and Narayan [37,38] predictability test
Name and reference of original method:	Westerlund, J., and Narayan, P.K., (2015). Testing for Predictability in Conditionally Heteroskedastic Stock Returns. Journal of Financial Econometrics, 13 342–375, https://doi.org/10.1093/jjfinec/nbu001 Westerlund, J., Narayan, P.K., (2012). Does the choice of estimator matter when forecasting returns? *Journal of Banking and Finance* 36, 2632–2640. https://doi.org/10.1016/j.jbankfin.2012.06.005
Resource availability:	*NA*

## Method details

### Introduction

In this note, we demonstrate the applicability of the Westerlund and Narayan (WN, [37,38]) predictability approach by considering COVID-19 and oil price data as predictors of stock market returns for four Asian countries, namely, Japan, Russia, Singapore, and South Korea (S. Korea).^1^ The motivation for using COVID-19 related data (total number of cases and total number of deaths) as predictors of stock returns has roots in the growing literature which shows that COVID-19 has had a significant effect on global economic and financial system which includes stock markets (see for instance, [12,18]; and [2], [3], [8], [11], [20], [39]). Moreover, some studies also demonstrate that unanticipated information (includes economic policy uncertainty, events such as terrorism and government shutdowns and the pandemic COVID-19) plays an important role in predicting stock returns (see [28]) and exchange rate returns (see for instance, [14,^23^,35]).

Inspired by these studies, we examine whether unanticipated events such as the COVID-19 pandemic can help us understand the predictability of stock returns during the volatile pandemic period. The negative sentiments or panic associated with disease outbreaks could have a substantial impact on financial markets (see [1,^5^,^7^,^16^,^17^,^20^,33]). In fact, a study by Haroon and Rizvi [9] shows that sentiments generated by COVID-19 related news lead to stock market fluctuations. Based on this literature, we hypothesize that the COVID-19 pandemic may contain valuable information to understand stock market returns.

The choice of the WN predictability approach is chosen because it accommodates three salient features of time-series data, namely persistency, endogeneity, and heteroskedasticity. If these data issues are not addressed, then it can lead to biased predictability results (see for instance, [24,^25^,36]). There are a number of studies which has already shown that the COVID-19 pandemic is volatile and any data related to COVID-19 is likely to be characterized by persistency (see [6,^10^,^15^,^19^,^22^,[31], [32], [33], [34] and heteroskedasticity (see for example, [15,33]). Thus, we believe the applicability of the WN approach to understanding the predictability of stock returns using time-series data is important for future researchers.

The balance of the paper is structured as follows. In the next section, we provide step-by-step predictability test approach. Section III provides a discussion on the application of the predictability model followed by some concluding remarks in the final section.

### Step-by-Step method

The following steps are followed in order to examine the time-series predictability using the WN approach.

Step 1: Implement a predictive regression model, where the dependent variable (denoted by $Y_{t}$) is regressed on the one-period lagged (or past information) predictor variable (denoted by $X_{t-1}$), which can be written as follows:

(1) $$Y_{t}=\;\propto +\beta X_{t-1}+e_{Y,t}$$

Step 2: It is possible that in step 1, the predictor variable is endogenous. If it is, it needs to be controlled by estimating the following regression:

(2) $$X_{t}=\mu \left(1-\gamma \right)+\gamma X_{t-1}+e_{X,t}$$

Where $e_{X,t}$ is mean zero and with variance $\sigma_{X}^{2}$.

Step 3: Test for endogeneity by extracting residuals from Eqs. (1) and (2). We assume that the error terms are linearly related and can be estimated in the following way:

(3) $$e_{Y,t}=\theta e_{X,t}+\varepsilon_{t}$$

Here, we are testing the null hypothesis of no endogeneity by setting θ=0 against an alternative that θ≠0.

Step 4: Estimate the final WN model, which uses two estimators, bias-adjusted ordinary least squares (OLS) and the generalized least squares (GLS). Both these estimators are based on Eq. (1) which is conditional on Eq. (2). This process removes the effect of the endogeneity through accommodating θ and persistency is accounted for by accommodating γ; therefore, the resulting conditional predictive regression model that is finally estimated is as follows:

(4) $$Y_{t}=\;\propto -\theta \mu \left(1-\gamma \right)+\beta^{adj}X_{t-1}+\theta \Delta X_{t}+\varepsilon_{t}$$

Here, we show that by construction $\varepsilon_{t}$ is independent of $e_{X,t}$ and $\beta^{adj}=\;\beta -\theta \left(\gamma -1\right)$.

Step 5: Control for heteroskedasticity by using the GLS estimator instead of using the OLS estimator. Therefore, it is assumed that $\varepsilon_{t}$ has the following autoregressive conditional heteroskedastic (ARCH) structure:

(5) $$\;\sigma_{\tau ,t}^{2}=\omega_{t}+\sum_{i=1}^{p}\omega_{i}\varepsilon_{t-i}^{2}$$

WN (2015) show that the conditional variance of $\varepsilon_{t}$ in Eq. (5) is $var\left(\varepsilon_{t}I_{t-1}\right)=\sigma_{\varepsilon ,t}^{2}=\theta^{2}\sigma_{\varepsilon ,t}^{2}=\sigma_{\tau ,t}^{2}$. Thus, the GLS *t*-statistics will have the following form:

(6) $$t_{GLS}=\frac{{\hat{\beta }}_{GLS}}{1/\sqrt{\sum_{i=2}^{T}\varphi_{j}^{2}{\left(X_{t-1}^{b}\right)}^{2}}}$$

Here, ${\hat{\beta }}_{GLS}$ is the GLS estimator of β from Eq. (5), $\varphi_{j}=1/\sigma_{Y,t}$ is the GLS weight, and $X_{t}^{b}=X_{t}-\sum_{k=2}^{T}X_{k}/T$, where T is the sample size. Hence, this can be simply interpreted as: the test statistic is dependent on endogeneity and persistency, which is the estimate of beta whereas heteroskedasticity appears in the denominator (which is the inverse of the standard deviation of adjusted dependent variable).

Step 6: Obtain $\beta^{adj}$ from Eq. (4), which denotes the coefficient of predictor variable.

Step 7: Test the null hypothesis of no predictability by setting $\beta^{adj}=0$ against the alternative that $\beta^{adj}\neq 0.$

## Application to COVID-19 and oil price data

### Data

We begin by providing a brief note on our dataset. Our dataset is daily and covers the sample 31/12/2019 – 01/12/2020. We consider stock indices for four Asian economies, namely Nikkei 225 stock index (Japan), the MOEX Russia index (Russia), the Straits Times index (Singapore), and the Korea Se Composite price index (S. Korea). All price data are sourced from DataStream. Using these stock market indices, we compute stock market returns as $log(SP/SP_{t-1})*100$. The West Texas Intermediate oil price (*OP*) is obtained from the US Energy Information Administration website (see https://www.eia.gov/dnav/pet/hist/RWTCD.htm). We use two proxies for COVID-19, namely the total number of cases (*TC*) and the total number of deaths (*TD*) caused by COVID-19. Daily data for both COVID-19 proxies are downloaded from our world in data website (see https://ourworldindata.org/grapher/daily-cases-covid-19).

As mentioned earlier in Section II, it is important to understand some common salient features of time-series data in order to make a better choice of the predictability methodology. Thus, the emphasis here is to understand the degree of persistency of the predictor variable, whether the predictor variable is endogenous, and whether the predictive regression model is heteroskedastic.^2^ We do not tabulate these results in this paper due to space constraint.^3^ Overall, we conclude that *TC* and *TD* are strongly persistent and heteroskedastic but not endogenous while *OP* is strongly persistent and endogenous but not heteroskedastic. This implies the need for addressing these issues in estimating the predictive regression model and our choice of the WN procedure is more ideal as it jointly accounts for all three salient features of time-series data.

### Predictability test results

In Table 1, we report predictability test results. In addition to using WN approach, we have also estimated our predictive regression model using OLS. Results obtained using OLS do not control for persistency, endogeneity and the heteroskedasticity in our predictive regression model. Thus, this implies that the findings obtained using the OLS model is biased and inefficient. This is very evident as for all predictor variables, the *t*-statistics reported in parenthesis for the OLS model are greater than those obtained using the WN approach. In order words, our findings indicate that if we do not control for persistency, endogeneity, and heteroskedasticity then it is easier to reject the null hypothesis of no predictability. This is what we show especially in the case of predictor variable, *TD*, which significantly predicts stock market returns of all four Asian countries using the OLS model. However, when we address these salient features of time-series data and predictability model using the WN approach, the value of *t*-statistics decreases, and overall, we conclude that COVID-19 does not significantly predict stock returns of Japan, Russia, and Singapore. The only exception is South Korea, where we find both proxies of COVID-19 to be statistically significant predictors of stock returns. Additionally, our results for predictor variable, *OP*, is consistent with results obtained using predictor variables, *TC* and *TD*.

**Table 1:** Predictability test results.

	*TC*		*TD*		*OP*	
Country	OLS	WN	OLS	WN	OLS	WN
Japan	0.0000 (1.5860)	0.1141 (1.3485)	0.0002* (1.8932)	0.0979 (1.6399)	−0.0041 (−0.6247)	−0.0562 (−1.0132)
Russia	0.0000 (1.2685)	−0.0238 (−0.2205)	0.0000 (1.1739)	−0.0130 (−0.1179)	0.0051 (0.7543)	0.0182 (0.7427)
Singapore	0.0000 (1.6075)	0.0889 (1.6221)	0.0107* (1.8097)	0.1056 (1.9356)	0.0040 (0.6519)	0.0149 (0.2693)
S. Korea	0.0000 (1.3109)	0.1012* (1.7403)	0.0009* (1.9062)	2.8648* (1.8651)	−0.0031 (−0.4286)	−0.0312 (−0.5596)

This table reports predictability test results obtained from estimating the predictability model proposed by WN [37,38].

* denotes statistical significance at the 10% level.

## Conclusion

This note demonstrates a step-by-step approach of the WN [37,38] predictability test. Future researchers will find this approach useful in order to examine the predictability of not only stock returns, but also exchange rate returns, commodity returns, and as well as predicting macroeconomic variables, such as change in gross domestic product (GDP), interest rates, and inflation rates. Most of these variables suffer from statistical issues of persistency, endogeneity and heteroskedasticity, which makes the WN model an ideal predictability tool.

## Declaration of Competing Interest

The authors declare that they have no known competing financial interests or personal relationships that could have appeared to influence the work reported in this paper.

## Footnotes

1 The literature which examines the connection between stock market and oil price (see for example, Narayan and Sharma 2011) is enormous, and therefore, we do not elaborate details of this literature in this note. Additionally, even though we do not examine the connection between oil price and the COVID-19 pandemic, it is important to acknowledge that this strand of literature is also rapidly growing (see [4], [7], [13], [17], [21], [28], [29], [30]).

2 The persistency of time series financial data can be determined using structural break unit root tests (see for instance, [26], [27]).

3 Detailed information on how we have examined persistency, heteroskedasticity and endogeneity tests and tabulated results are available upon request from the corresponding author.
